# Multi-transcript profiling in archival diagnostic prostate cancer needle biopsies to evaluate biomarkers in non-surgically treated men

**DOI:** 10.1186/1471-2407-14-673

**Published:** 2014-09-16

**Authors:** Naveen Kachroo, Anne Y Warren, Vincent J Gnanapragasam

**Affiliations:** Translational Prostate Cancer Group, Hutchison/MRC research centre, University of Cambridge, Hills Road, CB1 0XZ Cambridge, UK; Department of Pathology, Addenbrookes Hospital, Cambridge, UK

## Abstract

**Background:**

Most biomarkers in prostate cancer have only been evaluated in surgical cohorts. The value of these biomarkers in a different therapy context remains unclear. Our objective was to test a panel of surgical biomarkers for prognostic value in men treated by external beam radiotherapy (EBRT) and primary androgen deprivation therapy (PADT).

**Methods:**

The Fluidigm® PCR array was used for multi-transcript profiling of laser microdissected tumours from archival formalin-fixed diagnostic biopsies of patients treated by EBRT or PADT. Cases were matched for disease characteristics and had known 5 year biochemical relapse outcomes (n = 60). Results were validated by immunohistochemistry in a custom needle biopsy tissue microarray. Six biomarkers previously tested only in surgical cohorts were analysed (PTEN, E-Cadherin, EGFR, EZH2, PSMA, MSMB). Transcript and protein expression was correlated with clinical outcome analysed using Kruskal Wallis, Fisher’s test and Cox proportional hazard model.

**Results:**

Altered expression of E-Cadherin (p = 0.008) was associated with early relapse after EBRT. In PADT treated men however only altered MSMB transcript was prognostic for early relapse (p = 0.001). The remaining biomarkers however did not demonstrate prognostic ability in either cohort. In a separate tissue array we validated altered E-Cadherin protein as a predictor of early relapse after EBRT (n = 47) (HR 0.34, CI p = 0.02) but not in PADT treated men (n = 63).

**Conclusion:**

We demonstrate proof of principle of multiple transcript profiling in archival diagnostic biopsies of non-surgically treated men for biomarker discovery. We identify a role for E-Cadherin as a novel biomarker of early relapse following EBRT.

## Background

Clinical prostate cancer can be effectively treated by different modalities including surgery and external beam radiotherapy (EBRT) [1, 2]. There is currently no way of accurately predicting which therapy is best for an individual patient who may be otherwise eligible for both modalities [2, 3]. Tissue biomarkers that can predict and discriminate therapy outcome would therefore be an important and clinically useful tool. A critical issue with prostate cancer biomarker research is the amount of tissue available for analysis in non-surgically treated patients. As a result, the vast majority of biomarkers have only been tested and validated in surgically treated men [3]. For non-surgical therapies however the diagnostic needle biopsy is commonly the only tissue available to investigate potential biomarkers. Standard immunohistochemistry is not practical as a biomarker discovery platform because of the limited tissue available and significant heterogeneity in the biopsies. Moreover, only one candidate gene can usually be tested at a time.

Work in our group has developed methodology for multi-gene transcript profiling from laser micro-dissected formalin fixed paraffin embedded archival diagnostic needle biopsies [4–6]. In addition, we have recently undertaken a detailed systematic analysis of the available literature on tissue biomarkers within different therapy contexts [3]. This work has shown that there is a significant gap in the evidence for the usefulness of surgical biomarkers in other therapy contexts. In this study we bring together these two strands of work and utilise transcript profiling of laser micro-dissected diagnostic biopsies to test the usefulness of a panel of biomarkers which have not been hitherto tested in EBRT and/or primary androgen deprivation treated (PADT) cohorts. Our principal aim is to test the principle of simultaneously evaluating multiple surgically derived biomarkers in biopsies of men treated by non-surgical therapies and investigate if these will still retain prognostic ability.

## Methods

### Clinical database and transcriptome bank

Patients treated by either external beam radiotherapy with neo-adjuvant androgen deprivation (EBRT) or primary androgen deprivation therapy (PADT) only and with 5 year complete follow up data were identified from our hospital registry based at Addenbrookes Hospital, Cambridge. The study was conducted under specific ethical approval (Cambridgeshire 2 Research Ethics Committee, ethics 09/H0308/42). Men who had prolonged androgen deprivation after EBRT (more than 6 months) were excluded from the study. From these, men with and without early biochemical relapse were identified and included into age and tumour characteristic matched cohorts (n = 30 in each treatment cohort, 15 relapse and non-relapse in each). To achieve matching data was extracted from a cohort of men identified from the hospital pathology/clinical registry and we acquired relapsed cases first until we reached our stated numbers. Then we acquired matched selected non-relapse cases until we had the necessary cohort size. In any instance where there was more than 1 suitable case we selected the one with the closet match for the tumour characteristics. Biochemical relapse was defined for each treatment modality based on the EAU guidelines for prostate cancer [7]. For EBRT this was a PSA value of 2 ng/ml above the nadir. For PADT this was three consecutive rises of PSA, 1 week apart, resulting in two 50% increases over the nadir. The cohort size was derived based on *a priori* sample size calculation with advice from in house statisticians (University of Cambridge resource). Based on our previous PCR based expression studies in formalin fixed paraffin embedded tissue, we had established that at least a 2 fold change in gene expression was necessary for a significant difference. At a 90% power to detect a 2 fold difference at the 1% level of significance, the sample size required would be at least 20 in each treatment group (10 for each outcome). The present cohort size (30 in each group) is therefore sufficient for this analysis particularly as each variable would be considered independently.

The archived formalin fixed paraffin embedded (FFPE) diagnostic needle biopsy tissue for each case was acquired and all tumour areas in the tissue defined by a uro-pathologist (AW) by marking on matching H&E slides. All tumour areas were then laser capture microdissected and RNA extracted using a FFPE optimised protocol as previously published [4–6]. cDNA was synthesised (Transcriptor, Roche Diagnostics) and pre-amplified using specific target amplification. Briefly, equal volumes of 20× Taqman gene expression assay (see below for primers) were combined in a pooled assay mix. For each sample 1.25 μl cDNA (12.5 ng cDNA), 1.25 μl pooled assay mix and 2.5 μl Taqman PreAmp Master Mix (Applied Biosystems) was combined. These samples then underwent a thermal cycle programme of 95°C for 10 minutes, 14 cycles of 15 seconds at 95°C and 4 minutes at 60°C. The pre-amplified products were diluted to a 1:5 concentration in TE buffer. As a quality control step primers for 3 housekeeping genes were also included (β actin, GAPDH, RPL13) in the pre-amplification mix and tested by real time PCR.

### Biomarker panel

Candidate biomarkers were identified from a recent systematic review and had shown prognostic value in surgical cohorts but had not been tested in other therapies: E Cadherin, EGFR, EZH2, PTEN and MSMB [3]. These markers are also exemplars of biological events that are critical to prostate cancer progression (metastasis, growth factor signalling, transcription factor, cell survival and inhibitor of prostate cancer growth). We also included 3 highly prostate and prostate cancer specific genes as expression controls. The prostate marker PSMA which has shown promise as a prognostic marker following surgery but has not been tested in other treatment cohorts [8, 9]. We also included the androgen receptor (AR) which has not been tested in EBRT therapy as well as the generic marker prostate cancer antigen 3 (PCA3) [3, 10, 11]. All three were also selected as they are very well described genes expressed in prostate tissue and in prostate cancer. Taqman primers (Applied Biosystems) with the shortest amplicons lengths for these genes were acquired for this study: E Cadherin: Hs01023894_m1, EGFR: Hs01076078_m1, EZH2: Hs00544833_m1, PTEN: Hs02621230_s1, MSMB: Hs00738230_m1, amp length 72 PSMA: Hs01020194_mH, AR: Hs00171172_m1, PCA3: Hs01371939_g1, B-actin: Hs01060665_g1, GAPDH: Hs03929097_s1, RPL13: Hs00742932_s1, amp length 81.

### Fluidgm chip and quantitative real time PCR

The Fluidigm® 96.96 Dynamic Array integrated fluidic circuit chip was used to simultaneously profile the 9 gene panel (including β actin) in the 60 tumours as well as 2 benign prostate samples, 1 cancer line (PC3), 1 benign cell line (PNT2), a RNA positive control (Clontech, CA, USA) and a negative (water) control. Aliquots of each Gene Expression Assay were made up to a 10x concentration [2.5 μl of 20X Taqman Assay (Applied Biosystems) and 2.5 μl 2X Loading Reagent (Fluidigm®)]. For each tumour sample 2.5 μl Taqman Universal PCR Master Mix (Applied Biosystems) was combined with 0.25 μl 20X GE Sample Loading Reagent (Fluidigm® ) and 2.25 μl of the previously pre-amplified cDNA. Samples and assays were inputed into the appropriate inlets and run on the integrated fluidic controller to load the chip. The chip was then run on the Biomark Real Time PCR System using a cycling programme of 10 minutes at 95°C, 40 cycles of 95°C for 15 seconds and 1 minute at 60°C. Data was analysed using BioMark Gene Expression Data software to obtain Ct values and delta Ct values (corrected for β actin). Results shown are the mean of 3 assays which was replicated twice. Results were analysed statistically using the Kruskal Wallis test.

### Needle biopsy tissue microarray (TMA)

A separate cohort of EBRT and PADT cases were identified and for which sufficient archival FFPE tissue from diagnostic biopsies were available. Cases were again stratified as early biochemical relapse or no-relapse and age/tumour matched as described for the Fluidgm chip cohort above. The biopsy cores to be sampled from the donor blocks were marked on the corresponding Haematoxylin and Eosin stained paraffin sections by a consultant uro-histopathologist (AYW). These were selected by identifying representative tumour containing cores and which were used to ascribe the original tumour grade and extent for each case. 2 mm cores were punched from a selected area of the donor block using a disposable skin biopsy punch. The 2 mm punches were melted at 60°C to remove the excess wax and the donor cores embedded in the recipient paraffin block, lined with a thin cellulose template to act as a guide and to ensure that the cores from each case remained separated from one another. Wherever possible the cores were orientated at 90° to its neighbour which aided orientation during histological examination. Core positions in the recipient paraffin block were noted on a TMA map and a 2 mm pig kidney core was used as a marker for orientation. Three micron sections were cut and used for immunohistochemistry.

### Immunohistochemistry and scoring

Mouse monoclonal Ki67 and E Cadherin (Leica Biosystems, UK) antibodies have been previously validated [12, 13]. The Ki67 staining index was defined as the percentage of tumor cells that displayed positive nuclear staining per high powered field. A 7.1% cut off was used as previously described in radiotherapy immunohistochemistry studies with scores averaged across 4 different fields per section [14]. Immunoreactivity signals for E Cadherin were assessed as being absent or weak (0/+) and moderate or strong (++/+++). Scoring was done by two independent observers (AW&VG) blinded to the clinical detail and the scores collated. Discordant scores were reviewed jointly and rescored. Expression was compared between outcome groups using Fishers exact test. Data for E Cadherin in EBRT treated men was further analysed together with clinical variables in a Cox proportional hazards model for EBRT therapy. *p <* 0*.*05 was considered as statistically significant.

## Results

### Transcript expression of biomarker panel in biopsies linked to clinical outcomes

**Figure 1:**
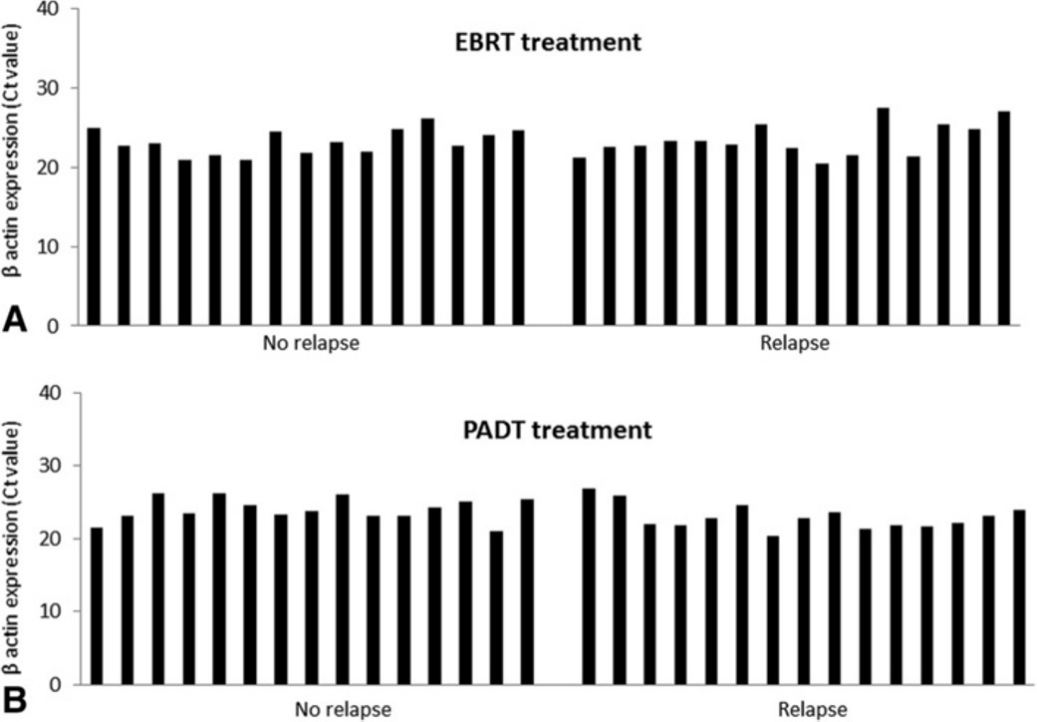
**Expression of housekeeping genes (β actin showed here) in laser micro-dissected individual tumours from archival formalin fixed paraffin embedded diagnostic biopsies from men treated by A. External beam radiotherapy (EBRT) B. Primary androgen deprivation therapy (PADT).**

**Figure 2:**
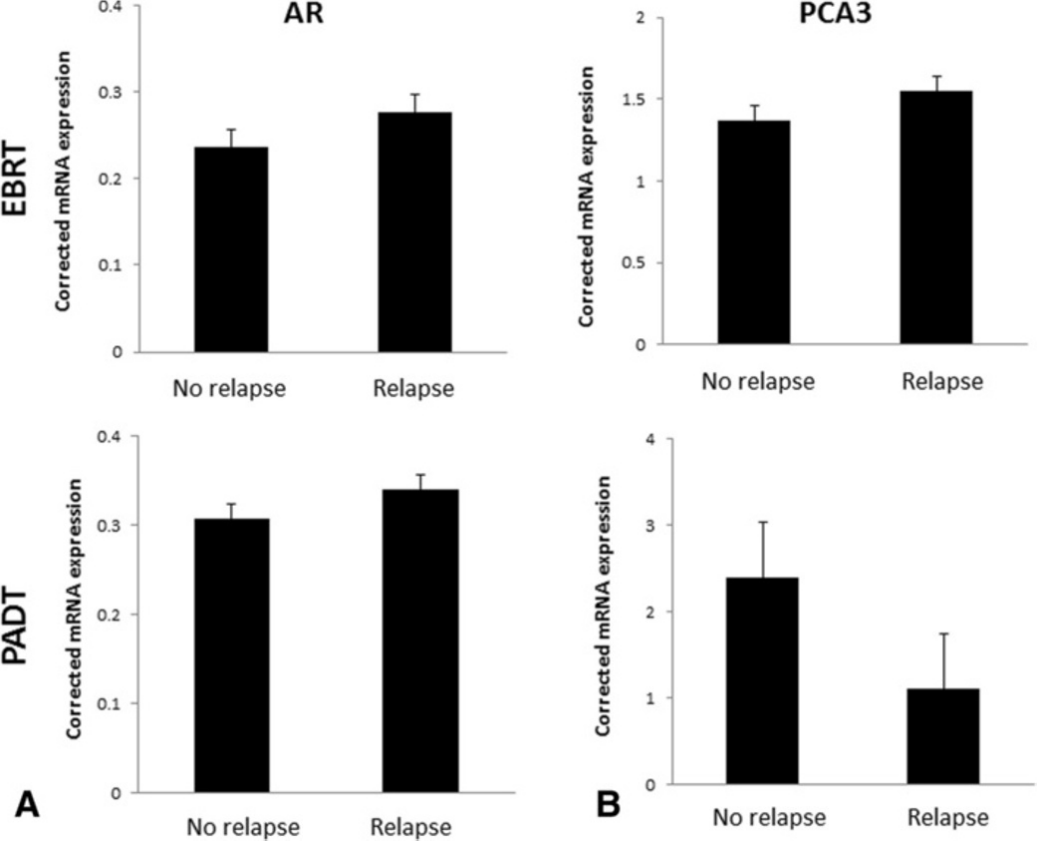
**Pooled transcript expression corrected to β actin micro-dissected archival FFPE diagnostic biopsies of men treated by EBRT or PADT and stratified by early biochemical relapse or no-relapse. A**. Expression of AR **B**. Expression of PCA3.

**Figure 3:**
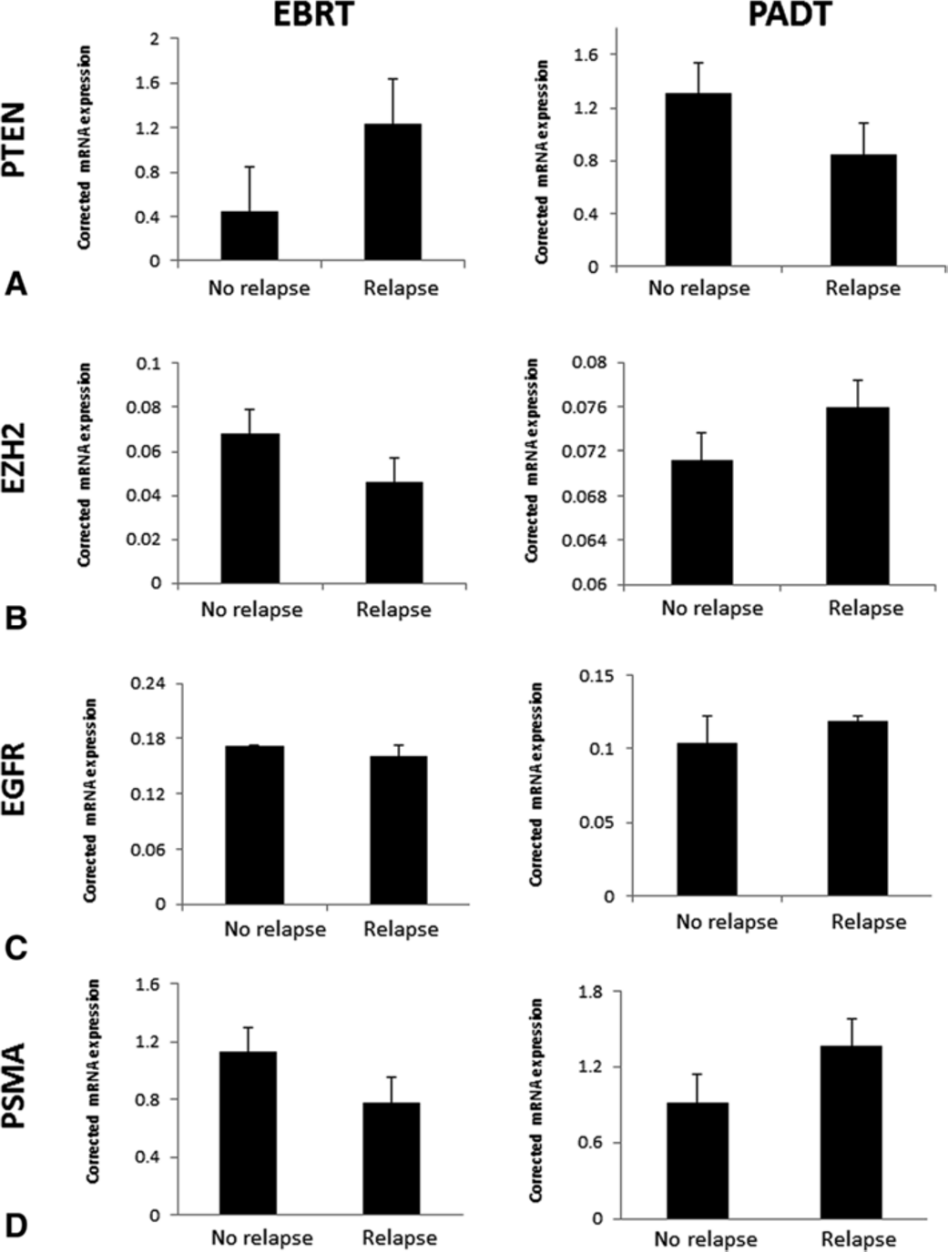
**Pooled transcript expression corrected to β actin in micro-dissected archival FFPE diagnostic biopsies of men treated by EBRT or PADT and stratified by early biochemical relapse or no-relapse. A**. Expression of PTEN **B**. Expression of EZH2. **C**. Expression of EGFR. **D**. Expression of PSMA.

**Figure 4:**
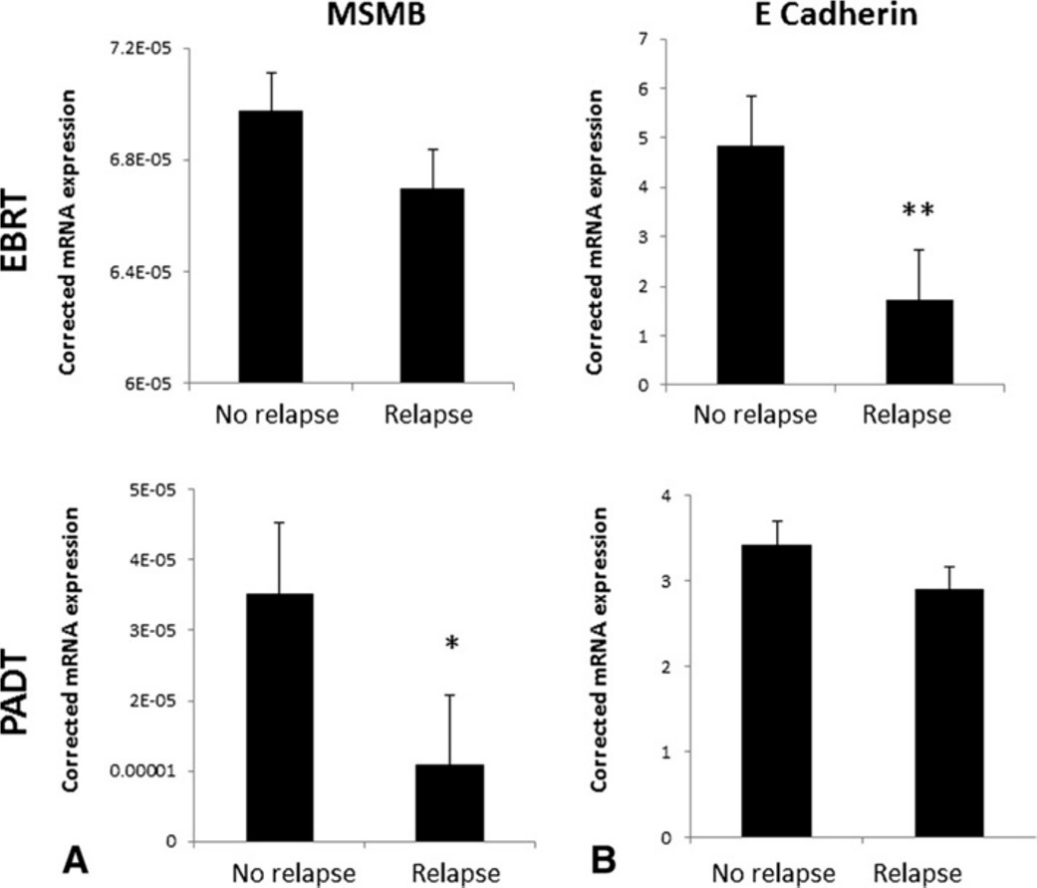
**Pooled transcript expression corrected to β actin micro-dissected archival FFPE diagnostic biopsies of men treated by EBRT or PADT and stratified by early biochemical relapse or no-relapse. A**. Expression of MSMB **B**. Expression of E Cadherin (*p = 0.02, **p < 0.01).

The baseline clinico-pathological features of the matched biochemical relapse and no-relapsed tumours included in the Fluidigm® array are shown in Table 1. RNA from all micro-dissected tumour samples were quality control checked by real time PCR for a panel of house-keeping genes using a previously reported method with good results (data not shown) [6, 15]. In addition, all samples were rechecked for housekeeping gene expression in the Fluidigm® array chip (Figure 1). We first assessed expression of the AR and PCA3. AR expression was not associated with good or poor outcomes from either EBRT or PADT treated cohorts (p = 0.49 and p = 0.75 respectively) (Figure 2A). Similarly, we did not observe any correlation between PCA3 expression and outcome from EBRT or PADT in this study (Figure 2B). We next assessed expression of the 5 surgical biomarker panel and PSMA. PTEN expression has been strongly associated with outcome in surgically treated men [16, 17]. In this study however PTEN expression was not associated with good or poor outcomes from either EBRT or PADT treatment (p = 0.54 and p = 0.34 respectively) (Figure 3A). We similarly found that mRNA expression levels of EZH2, EGFR and PSMA were also not statistically associated with good or poor outcomes in EBRT or PADT treated cohorts (Figure 3B-D). Reduced expression of MSMB has been shown to be associated with a poor outcome from surgery but has not been tested in EBRT cohorts. In this study MSMB had no prognostic value in EBRT treated men (p = 0.93) (Figure 4A). We did however find that MSMB expression was lower in men who had a poorer outcome from PADT. Of note, although the PADT groups in this study did not show statistical differences in the clinico-pathological characteristics there were more high grade, stage and metastasis cases in the relapse group (Table 1). Thus, a larger sample size may not detect this difference and this warrants further validation.

**Table 1:** **Baseline demographic data on disease characteristics of the cohort used in the transcript expression analysis stratified by early biochemical relapse or no-relapse (EBRT - external beam radiotherapy, PADT - primary androgen deprivation therapy)**

	PADT No relapse	PADT relapse	p value	EBRT No relapse	EBRT relapse	p value
**Sample size**	n = 15	n = 15		n = 15	n = 15	
**Mean age**	74 (57-78)	69 (60-78)	*p = 0.09 NS*	65 (58-71)	67 (52-77)	*p = 0.26 NS*
**Mean presenting PSA (ng/ml)**	23.9 (2.5-90.3)	25 (5.2-251)	*p = 0.36 NS*	15.5 (4.1-27.9)	23.9 (3.6-107)	*p = 0.25 NS*
**Clinical stage**			*p = 0.06 NS*			*p = 0.89 NS*
T1	2	0		2	4	
T2	9	7		8	5	
T3	4	7		5	6	
T4	0	1		0	0	
**Principle Gleason grade**			*p = 0.17 NS*			*p = 0.78 NS*
Gleason 3	7	4		12	11	
Gleason 4	6	6		2	4	
Gleason 5	2	5		1	0	
**Gleason grade sum**			*p = 0.25 NS*			*p = 0.93 NS*
6	3	1		8	7	
7	7	4		5	7	
8	0	5		0	0	
9	4	4		1	1	
10	1	1		1	0	
**Metastasis**			*p = 0.2 NS*			*p = 1.00 NS*
M0	13	9		15	15	
M1	2	6		0	0

Loss of E Cadherin has been shown to be a strong predictor of surgical outcomes in a number of studies [18, 19]. In this study reduced E Cadherin mRNA was significantly associated with a poorer outcome in EBRT but not PADT treated cohorts (p = 0.008 and p = 0.26 respectively) (Figure 4B).

### Immuno-histochemical validation of novel targets

**Figure 5:**
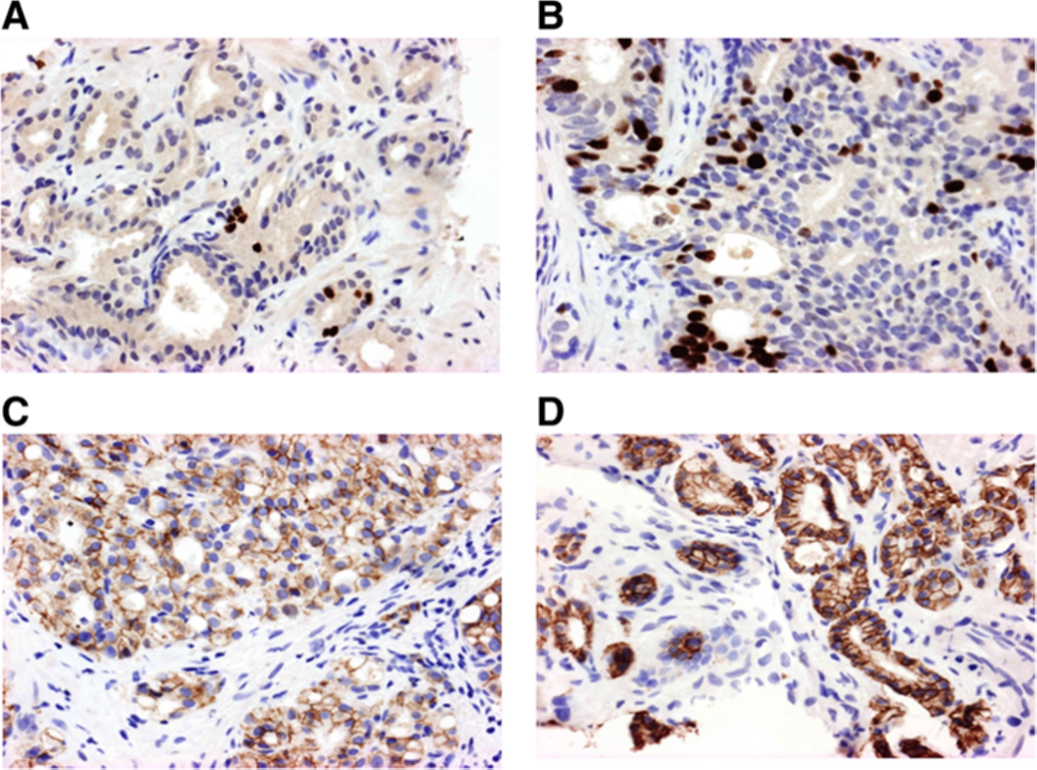
**Immunohistochemistry in diagnostic biopsies from EBRT treated men. A**. Low Ki67 expression **B**. High Ki67 expression. **C**. Low E Cadherin expression. **D**. High E Cadherin expression (X40 magnification for all images).

To further test the protein validity of our results with E Cadherin, we assembled a TMA of archival needle biopsy tissue from an extended EBRT and PADT cohorts identified from the clinical database and with known 5 year biochemical relapse outcomes. Tumours were again matched for grade, stage and presenting PSA. To test the robustness of this TMA we first interrogated for protein expression of the global prognostic marker Ki67. In both EBRT and PADT TMA, Ki67 was significantly over-expressed in the early relapse group compared to the non-relapse cohort using previously described scoring criteria (p = 0.0006 and p = 0.0004 respectively) (Figure 5A&B). These results are consistent with the published literature [20–22]. We next tested protein expression of E Cadherin in both groups using validated antibodies. In the EBRT cohort reduced E Cadherin protein was again significantly associated with early development of biochemical recurrence (p = 0.04) (Table 2) (Figure 5C&D). We further analysed the results in a Cox proportional hazards model including the clinical variables of presenting PSA, Gleason sum score and clinical stage. These variables were unsurprisingly not associated with outcomes as cases for this analysis had been matched for tumour characteristics. In this model however loss of E Cadherin was independently associated with an increase likelihood of early treatment failure (HR 0.34 [0.1-0.8] p = 0.02) (Table 3). In contrast E Cadherin protein expression was not associated with clinical outcome in PADT treated men. These data lend support to our initial observation at the transcript level of the prognostic value of E Cadherin expression for EBRT treated men but its lack of value in men treated by PADT.

**Table 2:** **Immunohistochemistry data on protein expression of Ki67 and E Cadherin stratified by early biochemical relapse or no-relapse**

**EBRT treatment**		**No relapse**	**Relapse**	
Ki67 (n = 54)	Low	26	4	
	High	9	15	p = 0.0004
E Cadherin (n = 47)	0**/+**	7	8	
	**++/+++**	25	7	p = 0.04
**PADT treatment**		**No relapse**	**Relapse**	
Ki67 (n = 53)	0**/+**	20	5	p = 0.0003
	**++/+++**	8	20	
E Cadherin (n = 63)	0**/+**	5	4	
	**++/+++**	20	34	p = 0.4

**Table 3:** **Cox proportional hazards model incorporating clinical variables and E Cadherin immune-staining scores in diagnostic biopsies as prognostic factors for early biochemical relapse for the external beam radiotherapy treated cohort**

	Hazard ratio	95% Confidence interval	p value
Presenting PSA	1.14	0.41-3.20	0.79
Clinical stage	0.79	0.21-3.01	0.73
Gleason sum	2.31	0.76-7.03	0.13
E Cadherin	0.34	0.13-0.89	0.02

## Discussion

The use of FFPE tissue for prognostic transcript profiling in prostate cancer is not new [23, 24]. Indeed, prognostic gene panels are about to enter mainstream commercial use for predicting prostate cancer therapy outcome [25, 26]. With reducing costs the era of massive parallel sequencing will soon be feasible for clinical use. However such platforms generally require good quality tissue input material and do not work so well on archival and FFPE tissue. PCR based assays however have been optimised to address this and new commercial tests have emerged that work well on prostate FFPE tissue [25]. Here we have sought to use PCR based FFPE transcript profiling to specifically test biomarkers in different therapy contexts and to our knowledge this approach is novel. Furthermore, our use of laser microdissected tumours dramatically increases the specificity and accuracy of profiling tumour cells without benign and stromal contamination [4, 27]. Using an FFPE optimised multiplex PCR platform we have been able to simultaneously analyse a number of biomarkers in well characterised treatment specific good and poor outcome cohorts.

This study has suggested preliminary evidence that loss of E Cadherin at the mRNA level is associated with a poorer outcome from EBRT but not in PADT treated men. We were further able to validate the results at the protein level in a custom made needle biopsy TMA. To our knowledge this is the first study to report on aberrant E Cadherin as a biomarker in EBRT for primary prostate cancer. One study in 2006 had reported that E Cadherin was a useful biomarker for patients who had subsequent salvage EBRT after RRP [28]. In this study however expression was profiled in the resected surgical sample and not the initial diagnostic biopsies. This is also the first study to explore E Cadherin in PADT treated men where no association was found with outcome at either the mRNA or protein level. The mechanistic rationale for the differential predictive ability of E Cadherin between EBRT and PADT in this study is intriguing. Recent studies have shown that EMT is associated with radioresistance in prostate cancer cells. Chang *et al* developed 3 radio resistant prostate cancer cell lines and demonstrated enhanced EMT phenotypes compared to controls [29]. Zhou *et al* have also previously shown that radiation therapy enhances the EMT process and ability of cells to migrate and invade [30]. Our work is the first to demonstrate the potential clinical association with EMT and radiotherapy outcome and lends support to these *in vitro* observations. If cancer cells have already acquired the EMT phenotype before radiation then it is more likely that this will promote micro-metastasis, radioresistance and early treatment failure. Loss of MSMB mRNA expression was however associated with early biochemical relapse in PADT treated men and is consistent with one older study has also previously shown that loss of expression was a predictor of a poor outcome in a mixed treatment group including men treated by primary androgen deprivation [31]. A number of other well known surgical biomarkers did not appear to have prognostic utility in different therapy contexts in our cohort. Work by Mucci and others have also previously shown this lack of transferability of biomarkers across therapies [32, 33]. We fully acknowledge that our discovery sample size is small (though matched and enriched for clinical outcomes) and we may have missed significant positive findings because of this. We therefore do not claim that our study rules out a role for the negative biomarkers tested.

The results of this study do raise important issues about biomarker discovery and use in non-surgically treated men. Prognostic ability in one context should not be extrapolated to other treatments without robust validation. This is a critical distinction if biomarkers are to be used to help therapy selection for patients and to guide future studies. The use of biomarkers in this context therefore must consider the treatment effect on cells and the mechanism of therapy resistance. Of note we had also found other surgical biomarkers in our previous review which had not been tested in EBRT or PADT cohorts [3]. Based on the encouraging results from this study we now intend to test these other markers further and within a rationale approach for therapy response and resistance. This work has shown the feasibility of testing multiple biomarkers in non-surgical cohorts. Clearly, this provides a very useful discovery platform but emergent markers need validation in independent and large cohorts. Optimised discovery methods however mean that such validation can be done using a targeted approach and with less resource intensive methods such as protein immunohistochemistry. Indeed we propose to further validate our current findings in larger cohorts from other collaborator centres and this work is underway. In the future testing fewer more relevant markers but in large datasets is the best route to rapidly developing clinically useful prognostic tools [34, 35].

There are a number of inherent limitations in this report. Our sample size (discovery and validation cohorts) although matched for clinic-pathological features, is relatively modest. The use of archival formalin fixed tissue is subject to mRNA degradation but we have applied optimised methodology for extraction as well as the use of short amplicons in PCR and well described quality control tests [4, 16]. We were not able to cross validate PCR and immunohistochemistry expression in the same sample as there was very limited amounts of tissue. However we were able to demonstrate the same results in separate cohorts which we believe is a strength of the study. We also acknowledge that needle biopsies may often underestimate the true disease burden in men treated by non-surgical therapy. However as previously discussed it is the only tissue that is ever available in EBRT and PADT treated men. Of note, the increasing use of template perineal and MRI guided prostate biopsies has already resulted in the real possibility of very accurate characterisation of the tumour burden from diagnostic needle biopsies alone and in men who will never have the prostate removed [36, 37]. The ability to perform transcript profiling in this context will become increasingly more relevant in identifying clinically useful biomarkers in non-surgical therapies. Finally, we were specifically interested in early biochemical relapse as a clinical outcome in this study. We are planning to follow the association of expression with metastasis and clinical progression outcome as the cohort matures.

## Conclusion

In summary we have applied multi-transcript profiling in well characterised cohorts to assess tissue biomarkers in archival FFPE tissue from non-surgically treated men. We show feasibility for simultaneous multiple biomarker testing in enriched tumour tissue from the original diagnostic needle biopsies as a platform for biomarker studies. This method would be particular useful to discover novel biomarkers specific to EBRT and other non-surgical therapies with the aim of targeted and rational validation in larger cohorts. In this context we demonstrate preliminary evidence for E Cadherin as a novel biomarker of EBRT outcome which warrants further investigation in larger multi-centre studies. Other biomarkers derived from surgical studies may not however have utility in a different therapy context suggesting that robust testing in appropriate cohorts is needed before inclusion in global prognostic models.
